# Quantitative evaluation of normal cerebrospinal fluid flow in Sylvian aqueduct and perivascular spaces of middle cerebral artery and circle of Willis using 2D phase-contrast MRI imaging

**DOI:** 10.3389/fnins.2025.1622351

**Published:** 2025-07-09

**Authors:** Rosemarie Faustina D. Le, Christof Karmonik, Angelique S. Regnier-Golanov, Eugene V. Golanov, Gavin W. Britz

**Affiliations:** ^1^Cerebrovascular Research Lab, Department of Neurosurgery, Houston Methodist Academic Institute, Houston Methodist, Houston, TX, United States; ^2^Translational Imaging Center, Houston Methodist Research Institute, Houston Methodist, Houston, TX, United States

**Keywords:** cerebrospinal fluid flow, Sylvian aqueduct, perivascular space, healthy volunteers, quantification, phase-contrast MRI, sex, age

## Abstract

**Introduction:**

Recently, it was proposed that CSF flow comprises a critical part of the glymphatic system, playing a role in various brain abnormalities from Alzheimer’s disease to hydrocephalus. Thus, CSF flow measurements have been increasingly used for diagnostic and clinical monitoring purposes. However, CSF flow in the periarterial spaces of the circle of Willis and the middle cerebral artery remain unexplored.

**Methods:**

We employed phase-contrast MRI to establish baseline parameters of CSF flow along the perivascular spaces of the circle of Willis and the middle cerebral artery and compare them with the Sylvian aqueduct. We also developed a new, semi-automated method for outlining the perivascular spaces and extracting CSF flow parameters. The 24 healthy participants were recruited to achieve an even distribution by age (mean: 40 ± 11) and gender (13 males, 11 females).

**Results:**

For most CSF flow parameters, the circle of Willis and middle cerebral artery were similar but differed from the Sylvian aqueduct. The linear mixed models and general linear mixed models for CSF flow parameters, except for time to peak velocity, indicated strong effects of the conduits. CSF velocity was lower by 0.159 cm/s in the circle of Willis and 0.198 cm/s in the middle cerebral artery than in the Sylvian aqueduct. Overall, differences in CSF flow parameters between sex and age groups were negligible.

**Discussion:**

Our semi-automated routine for CSF flow measurements in the Sylvian aqueduct (0.00700 mL/s) aligned with the range of literature values, 0.0049–0.0432 mL/s. In this study, we have established baseline values of CSF flow along the circle of Willis and the middle cerebral artery as well as highlighted the limited influence of sex and/or age.

## 1 Introduction

Cerebrospinal fluid (CSF) is a clear fluid surrounding the central nervous system and filling the cerebral ventricles and the central canal of the spinal cord. At any given time, there are 90–150 mL of CSF in the cranium and spinal cord because production and absorption occur at the same rate to maintain intracranial pressure (Sartoretti et al., 2019; Vandenbulcke et al., 2022). CSF is produced in the choroid plexus of the lateral ventricles; it flows through the interventricular foramina to the third ventricle, along the Sylvian aqueduct (SA) to the fourth ventricle, proceeding to the subarachnoid space, and arriving in the spinal cord (Sartoretti et al., 2019; Liu et al., 2022; Vandenbulcke et al., 2022). Recently, the concept of the glymphatic pathway has been introduced (Iliff et al., 2012; Hablitz and Nedergaard, 2021). In the glymphatic system, CSF flows from the subarachnoid space to the periarterial space and enters the brain parenchyma, where it mixes with the interstitial fluid and exits into the paravenous space. CSF flow is pulsatile due to cardiac pulsations and breathing (Mestre et al., 2018; Liu et al., 2022). Depending on the cardiac cycle, CSF moves in opposite directions. During systole, intracranial arteries increase in volume which pushes CSF in the craniocaudal direction. The reverse happens in diastole as blood vessel volume decreases and CSF moves in the caudocranial direction (Lee et al., 2004; Vandenbulcke et al., 2022; Rohilla et al., 2023).

CSF serves several physiological functions. CSF provides support and cushioning for the brain. Movement of CSF through the brain parenchyma clears out waste and transports essential molecules (Howden et al., 2008; Nedergaard, 2013; Sartoretti et al., 2019; Liu et al., 2022; Vandenbulcke et al., 2022). These functions and dynamics, especially CSF flow and pressure, can be altered in pathologies. As part of the glymphatic system, CSF flow has been proposed to play a role in Alzheimer’s disease by failing to clear out amyloid beta and tau tangles, the characteristic protein markers (Stoquart-ElSankari et al., 2007; Liu et al., 2022; Reeves, 2025). Abnormal increase in intracranial pressure due to abnormal CSF drainage is associated with hydrocephalus (Lee et al., 2004; Stoquart-ElSankari et al., 2007; Rohilla et al., 2023; Reeves, 2025). Other studies have observed the roles of CSF flow in meningitis, cerebral edema, and other cerebrovascular diseases (Stoquart-ElSankari et al., 2007; Rohilla et al., 2023). Thus, measurement of CSF flow has been increasingly used for diagnostic and clinical monitoring purposes (Rohilla et al., 2023).

To explore the clinical implications of CSF flow, many studies focused on CSF flow through the SA which connects the third and fourth ventricles (Howden et al., 2008; Sartoretti et al., 2019). SA is easier to identify and image, so it has been studied frequently. On the other hand, the periarterial spaces of the circle of Willis (COW) and the middle cerebral artery (MCA), important conduits of CSF flow, remain unexplored. The COW is localized to the base of the brain and formed by anterior cerebral arteries, anterior communicating artery, internal carotid arteries (at their distal tips), posterior cerebral arteries, and posterior communicating artery. MCA originates from COW at the internal carotid arteries, joining and ascending in the lateral sulcus of the cerebrum, providing blood supply to many parts of the lateral cortex.

Therefore, this study has several objectives. First, because the CSF flow along the perivascular spaces of COW and MCA has not been examined, we aim to establish baseline measurements of CSF flow parameters along these conduits, including potential differences due to sex and age. Second, we aim to compare COW and MCA CSF flow parameters to the well-established flow through the SA.

To analyze CSF flow, we developed a new, semi-automated method for outlining perivascular spaces and extracting CSF flow parameters (Supplementary Figure 1). Many semi-automated or automated methods have been developed in order to increase accuracy, save time on analysis, and/or contribute to study reproducibility (Flórez et al., 2006; Yoshida et al., 2009). We conducted preliminary evaluation of our semi-automated program through validation with CSF flow parameters in the SA which are extensively covered in the literature.

We focused on the following CSF flow parameters: stroke volume (StroVol), volumetric flow rate (VFR), systolic flow rate (SFR), diastolic flow rate (DFR), velocity, peak systolic velocity (PSV), and peak diastolic velocity (PDV). While the most useful parameters have yet to be determined, we chose these parameters to capture the overall picture of CSF flow dynamics in the hopes that they may be used for future clinical diagnostics and therapeutics (Wagshul et al., 2006).

## 2 Materials and methods

### 2.1 Participants

The 24 healthy participants were recruited to achieve an even distribution by age and gender (13 males, 11 females). Ages ranged from 23 to 59 with a mean of 40 ± 11 (Table 1). To obtain baseline parameters by demographics, participants were separated into different age groups: 20–29, 30–39, 40–49, and 50–59. The study was approved by the Houston Methodist Hospital Institutional Review Board, protocol Pro00022145.

**Table 1:** Participant demographics.

Demographics	Description	Number	Percentage
Age	20–29	6	25%
	30–39	4	17%
	40–49	7	29%
	50–59	7	29%
	Total	24	100%
Sex	Male	13	54%
	Female	11	46%
	Total	24	100%

### 2.2 MRI acquisition

For this study, we used phase-contrast magnetic resonance imaging (PC-MRI). PC-MRI was first used in Barkhof et al. (1994) to evaluate the effect of age on SA flow. Since then, PC-MRI has emerged as the gold standard for measuring CSF flow (Liu et al., 2024). The advantages of PC-MRI are it is noninvasive and relatively quick at taking measurements (Stoquart-ElSankari et al., 2007).

A semi-automatic analysis method was developed to streamline region selection and ensure consistent CSF flow quantification across subjects. This tool integrates anatomical reference points and threshold-based segmentation to minimize operator bias. Future studies may extend this approach to other anatomical regions and validate its robustness further.

A 2D PC-MRI image was acquired at each region of interest (SA and the perivascular spaces of COW and MCA) chosen by an experienced neuroscientist (EG, ARG). Details of the acquisition parameters are as follows: slice thickness 3 mm, acquisition matrix 272 × 272, FOV 160 cm 160 cm, in-plane resolution 0.6 mm x 0.6 mm. TE = 3 msec, TR = 105 msec. Images were acquired with a single transmit, 32 channel receive head coil in the FDA-approved clinical mode of the MAGNETOM 7 Tesla human MRI scanner (Siemens Healthineers, Erlangen, Germany). A 3D velocity encoding scheme was used to account for CSF flow direction not exactly parallel to the slice normal. The encoding velocity (VENC) was set at 5 cm/s to remain sensitive to the corresponding flow velocity of CSF flow and to eliminate blood flow contamination, recognized as global maximum/minimum gray scale values, due to the phase wrapping artifact of blood velocities exceeding this VENC value. A total of 20 images evenly spaced across the cardiac cycle were reconstructed with a total acquisition time of approximately 5 min for each location.

### 2.3 CSF flow analysis

As the VENC value was low compared to blood flow velocities, global maximum black/white gray scale intensity values—caused by the phase wrapping artifact—were used as a mask for distinguishing and eliminating blood flow from perivascular CSF flow in 2D phase contrast images. Regions of interest (ROIs) in an annular shape centered on the arterial cross section were chosen for quantifying CSF flow using a semi-automated algorithm described in Supplementary Figure 1.

For the first phase image of the 20 series of images for the cardiac cycle, the X, Y-coordinates, tolerance, and band size of the ROIs were determined by the user (Supplementary Figure 1).

**Figure 1:**
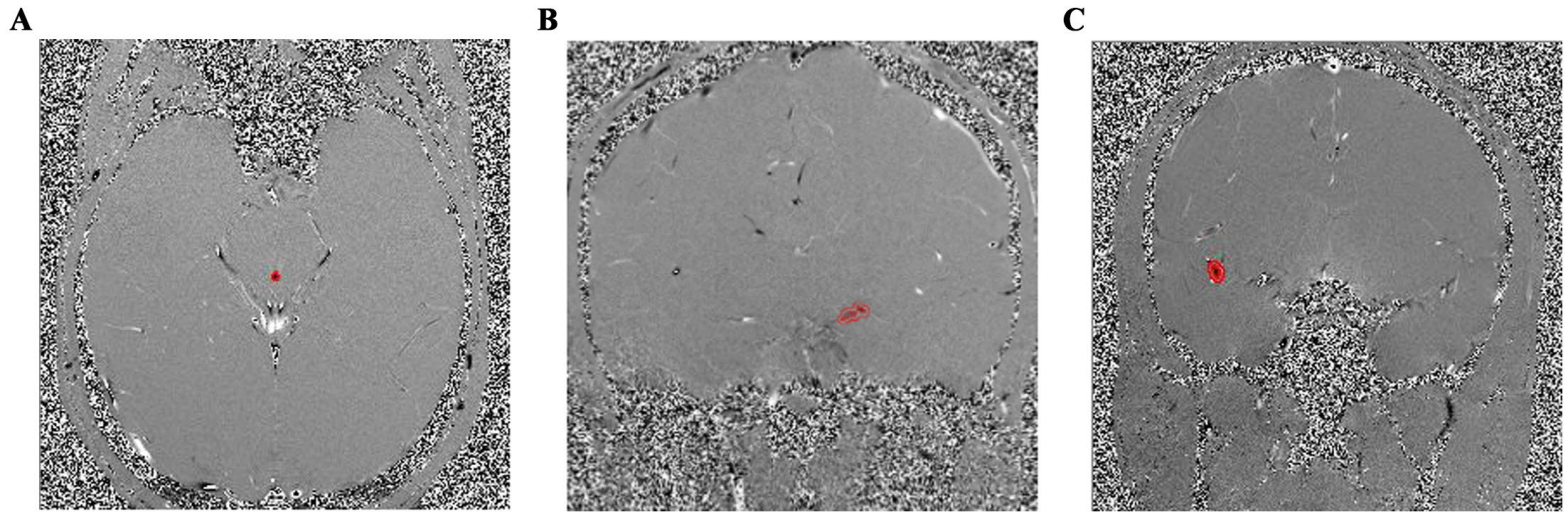
Examples of ROIs. **(A)** ROI of SA, **(B)** one of two ROIs of COW, **(C)** and one of two ROIs of MCA on phase images.

Magnitude images were used as anatomical references. Two ROIs—one left and one right—were identified for each COW and MCA, and one ROI was identified for SA (Figure 1). The established ROI settings were entered into our developed in-house semi-automated program to produce flow rate (FR) and Velocity values. FR (mL/s) was calculated as the $\text{Area of the}\;\mathrm{ROI}\;(\text{converted from}\;mm^{2}\;\text{to}\;cm^{2})\times \text{VENC}\times (\frac{\text{mean intensity}}{\text{maximum gray scale intensity}}).$ Velocity (cm/s) was calculated as FR÷area of theROI . ROIs for 20 cardiac gated images over one cardiac cycle were obtained from each series to obtain the FR and Velocity curves.

Peak timing was standardized by looking at which percentage of the cardiac cycle peak flow occurred. The percentage of the cardiac cycle was calculated as $(\frac{\text{the ordinal position of the image within the series}}{20})\times 100$ . The stroke volume was calculated as FR×time .

### 2.4 Statistical analysis

For all parameters in each conduit of SA, COW, and MCA, median values and bias-corrected and accelerated bootstrap confidence intervals (CIs) from non-parametric bootstrapping were calculated.

To compare the conduits and evaluate the influence of demographics, various models were constructed. For StroVol, VFR, and Velocity, linear mixed models (LMM) were constructed with block bootstrapping. Generalized linear mixed models (GLMM) with the gamma family and log link function were constructed with block bootstrapping for SFR, DFR, and Area. Generalized linear models (GLM) were constructed for PSV and PDV. For these LMMs, GLMMs, and GLMs, accelerated bootstrap CIs were calculated. For time to peak as percentage of cardiac cycle duration (TP) of StroVol (TPStroVol), VFR (TPVFR), and Velocity (TPVelocity), beta regression with the logit link function was constructed, and Wald CIs were calculated. For comparing SA, COW, and MCA, fixed effects were the intercept (= SA), time (except for PSV, PDV, and TP parameters), COW, and MCA. SA was set as the intercept because CSF flow parameters for the SA are already established in the literature. For evaluating the influence of sex and age, fixed effects were the intercept, time (except for PSV, PDV, and TP parameters), sex, and age. An interaction term between sex and age was also added. When applicable, participants were considered as random effects to account for the non-independence of time series. Age was rescaled, zero random effects were dropped, and/or optimizers were changed to address model convergence issues as needed. Whenever the data contained zero-values, the following transformation was applied to the outcome variable before beta regression: $\frac{\left(\text{outcome variable}\times 23+0.5\right)}{23}$ . For *post-hoc* testing, *p*-values were adjusted using the Benjamini-Hochberg procedure. Assumptions were checked with QQ plots and residual plots. All statistical analysis and visualization were performed in R/RStudio.

## 3 Results

Baseline values of CSF flow parameters with the median and 95% CI by conduits and by sex and age are reported in Tables 2–4, respectively. Model estimates of the fixed and random effects are reported in Table 5 for conduits comparison and Supplementary Table 1 for the effect of sex and age. StroVol is reported in mL. VFR, SFR, and DFR are reported in mL/s. Velocity, PSV, and PDV are reported in cm/s. TPStroVol, TPVFR, and TPVelocity are reported in percentage of the cardiac cycle. Area is reported in cm^2^.

**Table 2:** Median values of CSF flow parameters in SA, COW, and MCA.

	Conduit	Median	Lower 95% CI	Upper 95% CI
StroVol (mL)	SA	0.00254	0.00221	0.00277
	COW	0.0146	0.0140	0.0156
	MCA	0.0140	0.0131	0.0149
VFR (mL/s)	SA	0.00700	0.00600	0.00700
	COW	0.0420	0.0400	0.0430
	MCA	0.0450	0.0420	0.0460
SFR (mL/s)	SA	0.00900	0.00800	0.00900
	COW	0.0530	0.0500	0.0570
	MCA	0.0550	0.0530	0.0580
DFR (mL/s)	SA	0.00400	0.00400	0.00500
	COW	0.0230	0.0220	0.0250
	MCA	0.0270	0.0230	0.0280
Velocity (cm/s)	SA	0.578	0.578	0.723
	COW	0.285	0.271	0.299
	MCA	0.260	0.246	0.267
PSV (cm/s)	SA	1.45	1.16	1.45
	COW	0.749	0.677	0.848
	MCA	0.676	0.516	0.728
PDV (cm/s)	SA	0.867	0.578	0.867
	COW	0.383	0.368	0.622
	MCA	0.405	0.303	0.452
TPStroVol (%)	SA	50.0	40.0	62.5
	COW	75.0	70.0	80.0
	MCA	70.0	70.0	80.0
TPVFR (%)	SA	20.0	20.0	25.0
	COW	40.0	30.0	55.0
	MCA	40.0	15.0	42.5
TPVelocity (%)	SA	55.0	50.0	55.0
	COW	65.0	50.0	65.0
	MCA	65.0	55.0	75.0
Area (cm^2^)	SA	0.00692	0.00692	0.0104
	COW	0.0277	0.0277	0.0311
	MCA	0.0398	0.0381	0.0415

**Table 3:** Median values of CSF flow parameters in different conduits by sex.

	Conduit	Sex	Median	Lower 95% CI	Upper 95% CI
StroVol (mL)	SA	Male	0.00290	0.00258	0.00310
		Female	0.00196	0.00171	0.00219
	COW	Male	0.0157	0.0142	0.0173
		Female	0.0140	0.0126	0.0147
	MCA	Male	0.0140	0.0128	0.0152
		Female	0.0141	0.0128	0.0158
VFR (mL/s)	SA	Male	0.00800	0.00800	0.0100
		Female	0.00500	0.00500	0.00700
	COW	Male	0.0450	0.0425	0.0490
		Female	0.0380	0.0360	0.0410
	MCA	Male	0.0450	0.0430	0.0490
		Female	0.0440	0.0420	0.0470
SFR (mL/s)	SA	Male	0.0100	0.00800	0.0110
		Female	0.00700	0.00600	0.00900
	COW	Male	0.0580	0.0530	0.0620
		Female	0.0480	0.0460	0.0550
	MCA	Male	0.0590	0.0570	0.0640
		Female	0.0520	0.0490	0.0550
DFR (mL/s)	SA	Male	0.00500	0.00500	0.00700
		Female	0.00300	0.00200	0.00300
	COW	Male	0.0250	0.0220	0.0270
		Female	0.0220	0.0180	0.0230
	MCA	Male	0.0280	0.0230	0.0290
		Female	0.0235	0.0190	0.0295
Velocity (cm/s)	SA	Male	0.674	0.578	0.761
		Female	0.528	0.482	0.578
	COW	Male	0.324	0.299	0.347
		Female	0.256	0.241	0.275
	MCA	Male	0.260	0.241	0.271
		Female	0.260	0.246	0.275
PSV (cm/s)	SA	Male	1.59	1.45	1.88
		Female	1.30	0.867	1.45
	COW	Male	0.771	0.742	1.08
		Female	0.737	0.526	0.795
	MCA	Male	0.686	0.552	0.867
		Female	0.605	0.489	0.778
PDV (cm/s)	SA	Male	1.32	0.896	1.73
		Female	0.578	0.578	0.867
	COW	Male	0.420	0.310	0.688
		Female	0.383	0.171	0.506
	MCA	Male	0.415	0.348	0.518
		Female	0.330	0.282	0.487
TPStroVol (%)	SA	Male	55.0	40.0	65.0
		Female	35.0	25.0	60.0
	COW	Male	70.0	70.0	85.0
		Female	75.0	70.0	87.5
	MCA	Male	75.0	65.0	75.0
		Female	65.0	65.0	80.0
TPVFR (%)	SA	Male	20.0	15.0	25.0
		Female	20.0	17.5	42.5
	COW	Male	35.0	15.0	45.0
		Female	50.0	35.0	70.0
	MCA	Male	35.0	10.0	40.0
		Female	40.0	15.0	45.0
TPVelocity (%)	SA	Male	55.0	55.0	75.0
		Female	50.0	44.9	50.0
	COW	Male	65.0	50.0	65.0
		Female	70.0	50.0	80.0
	MCA	Male	70.0	60.0	80.0
		Female	62.5	45.0	75.0
Area (cm^2^)	SA	Male	0.00692	0.00692	0.0104
		Female	0.0104	0.00692	0.0104
	COW	Male	0.0277	0.0277	0.0329
		Female	0.0277	0.0242	0.0346
	MCA	Male	0.0450	0.0381	0.0450
		Female	0.0346	0.0311	0.0415

**Table 4:** Median values of CSF flow parameters in different conduits by age group.

	Conduit	Age group	Median	Lower 95% CI	Upper 95% CI
StroVol (mL)	SA	20–29	0.00217	0.00194	0.00256
		30–39	0.00288	0.00219	0.00407
		40–49	0.00303	0.00268	0.00338
		50–59	0.00190	0.00173	0.00285
	COW	20–29	0.0143	0.0115	0.0157
		30–39	0.0129	0.0110	0.0143
		40–49	0.0169	0.0156	0.0192
		50–59	0.0143	0.0128	0.0157
	MCA	20–29	0.0121	0.0102	0.0133
		30–39	0.0176	0.0145	0.0205
		40–49	0.0174	0.0160	0.0203
		50–59	0.0118	0.0108	0.0132
VFR (mL/s)	SA	20–29	0.00600	0.00600	0.00800
		30–39	0.00700	0.00600	0.00900
		40–49	0.00700	0.00700	0.00850
		50–59	0.00600	0.00500	0.00900
	COW	20–29	0.0440	0.0410	0.0480
		30–39	0.0335	0.0295	0.0370
		40–49	0.0450	0.0415	0.0480
		50–59	0.0390	0.0350	0.0450
	MCA	20–29	0.0375	0.0340	0.0420
		30–39	0.0545	0.0475	0.0600
		40–49	0.0530	0.0490	0.0560
		50–59	0.0370	0.0330	0.0420
SFR (mL/s)	SA	20–29	0.00800	0.00600	0.00900
		30–39	0.0100	0.00800	0.0140
		40–49	0.00900	0.00700	0.00900
		50–59	0.00800	0.00500	0.0100
	COW	20–29	0.0510	0.0460	0.0560
		30–39	0.0490	0.0390	0.0580
		40–49	0.0580	0.0530	0.0640
		50–59	0.0525	0.0470	0.0580
	MCA	20–29	0.0500	0.0430	0.0530
		30–39	0.0620	0.0553	0.0720
		40–49	0.0660	0.0590	0.0700
		50–59	0.0470	0.0430	0.0495
DFR (mL/s)	SA	20–29	0.00350	0.00300	0.00500
		30–39	0.00400	0.00200	0.00500
		40–49	0.00500	0.00300	0.00500
		50–59	0.00400	0.00200	0.00500
	COW	20–29	0.0260	0.0230	0.0330
		30–39	0.0200	0.0150	0.0230
		40–49	0.0260	0.0250	0.0330
		50–59	0.0210	0.0189	0.0240
	MCA	20–29	0.0270	0.0210	0.0290
		30–39	0.0285	0.0185	0.0455
		40–49	0.0310	0.0290	0.0360
		50–59	0.0190	0.0145	0.0210
Velocity (cm/s)	SA	20–29	0.556	0.455	0.578
		30–39	0.540	0.442	0.589
		40–49	0.674	0.578	0.783
		50–59	0.723	0.578	0.867
	COW	20–29	0.293	0.267	0.326
		30–39	0.242	0.200	0.264
		40–49	0.295	0.273	0.320
		50–59	0.289	0.270	0.328
	MCA	20–29	0.267	0.248	0.289
		30–39	0.273	0.248	0.318
		40–49	0.269	0.253	0.293
		50–59	0.237	0.217	0.255
PSV (cm/s)	SA	20–29	1.16	1.16	1.73
		30–39	1.30	1.30	2.31
		40–49	1.45	1.45	1.88
		50–59	1.59	1.16	3.47
	COW	20–29	0.674	0.462	0.694
		30–39	0.751	0.605	1.29
		40–49	0.751	0.418	0.771
		50–59	0.828	0.766	1.05
	MCA	20–29	0.535	0.484	0.815
		30–39	0.676	0.124	0.700
		40–49	0.766	0.489	0.867
		50–59	0.605	0.500	0.867
PDV (cm/s)	SA	20–29	0.867	0.247	0.867
		30–39	0.578	0.578	1.76
		40–49	1.07	0.867	1.54
		50–59	0.723	0.578	2.75
	COW	20–29	0.171	0.0942	0.650
		30–39	0.390	0.390	0.650
		40–49	0.376	0.239	0.824
		50–59	0.491	0.289	0.549
	MCA	20–29	0.405	0.348	0.518
		30–39	0.275	0.303	1.49
		40–49	0.500	0.303	0.590
		50–59	0.318	0.128	0.381
TPStroVol (%)	SA	20–29	57.5	28.7	67.5
		30–39	25.0	20.0	70.0
		40–49	50.0	25.0	65.0
		50–59	50.0	45.0	60.0
	COW	20–29	75.0	70.0	92.5
		30–39	70.0	70.0	90.0
		40–49	70.0	67.5	90.0
		50–59	75.0	50.0	75.0
	MCA	20–29	65.0	65.0	80.0
		30–39	65.0	65.0	90.0
		40–49	75.0	50.0	75.0
		50–59	70.0	70.0	90.0
TPVFR (%)	SA	20–29	17.5	10.0	35.0
		30–39	22.5	20.0	67.5
		40–49	22.5	12.5	25.0
		50–59	20.0	5.00	55.0
	COW	20–29	50.0	27.5	60.0
		30–39	42.5	32.5	70.0
		40–49	25.0	20.0	55.0
		50–59	50.0	20.0	60.0
	MCA	20–29	47.5	35.0	65.0
		30–39	45.0	32.5	65.0
		40–49	15.0	15.0	60.0
		50–59	35.0	15.0	45.0
TPVelocity (%)	SA	20–29	55.0	50.0	72.5
		30–39	50.0	50.0	90.0
		40–49	55.0	55.0	85.0
		50–59	50.0	40.0	55.0
	COW	20–29	60.0	35.0	75.0
		30–39	80.0	60.0	90.0
		40–49	57.5	50.0	72.5
		50–59	60.0	45.0	80.0
	MCA	20–29	60.0	45.0	90.0
		30–39	57.5	30.0	75.0
		40–49	75.0	45.0	80.0
		50–59	70.0	45.0	80.0
Area (cm^2^)	SA	20–29	0.0104	0.00692	0.0104
		30–39	0.0121	0.0104	0.0173
		40–49	0.0104	0.00692	0.0104
		50–59	0.00692	0.00346	0.00692
	COW	20–29	0.0311	0.0242	0.0346
		30–39	0.0311	0.0260	0.0415
		40–49	0.0311	0.0277	0.0381
		50–59	0.0208	0.0208	0.0293
	MCA	20–29	0.0311	0.0294	0.0381
		30–39	0.0588	0.0484	0.0813
		40–49	0.0484	0.0415	0.0519
		50–59	0.0346	0.0277	0.0381

**Table 5:** Estimates of models of CSF flow parameters in SA (intercept) vs. COW vs. MCA.

	Fixed effects	Coefficient	Lower 95% CI	Upper 95% CI	Random effects	Variance	SD	H0	*P*-value
StroVol	Intercept (SA)	−0.00857	−0.0124	−0.00527	Intercept: participants	5.22E-06	0.00228	SA = COW = MCA	0.000999
	Time	0.0278	0.0207	0.0335	Residuals	0.00142	0.0376	SA = COW	0.00400
	COW	0.0150	0.0103	0.0190				SA = MCA	0.00400
	MCA	0.0142	0.00979	0.0194				COW = MCA	0.805
VFR	Intercept (SA)	0.0230	0.0164	0.0303	Intercept: participants	2.24E-05	0.00474	SA = COW = MCA	0.000999
	Time	−0.0241	−0.0363	−0.0139	Residuals	0.00598	0.0773	SA = COW	0.00400
	COW	0.0350	0.0261	0.0460				SA = MCA	0.00400
	MCA	0.0310	0.0203	0.0411				COW = MCA	0.529
SFR	Intercept (SA)	−3.73	−3.88	−3.63	Intercept: participants	0.0172	0.131	SA = COW = MCA	0.00100
	Time	−0.483	−0.705	−0.320	Residuals	1.07	1.03	SA = COW	0.00400
	COW	1.36	1.20	1.50				SA = MCA	0.00400
	MCA	1.36	1.21	1.51				COW = MCA	0.969
DFR	Intercept (SA)	−4.78	−5.17	−4.55	Intercept: participants	0.0738	0.272	SA = COW = MCA	0.00100
	Time	−0.536	−0.802	−0.245	Residuals	0.816	0.903	SA = COW	0.00799
	COW	1.48	1.16	1.83				SA = MCA	0.00799
	MCA	1.61	1.33	1.94				COW = MCA	0.216
Velocity	Intercept (SA)	0.421	0.318	0.534	Intercept: participants	0.00180	0.0424	SA = COW = MCA	0.000999
	Time	−0.104	−0.178	−0.0277	Residuals	0.308	0.555	SA = COW	0.0160
	COW	−0.159	−0.286	−0.0498				SA = MCA	0.00599
	MCA	−0.198	0.0204	0.0410				COW = MCA	0.166
PSV	Intercept (SA)	0.537	0.413	0.645				SA = COW = MCA	0.000999
	COW	−0.773	−0.954	−0.613				SA = COW	0.00400
	MCA	−0.955	−1.12	−0.773				SA = MCA	0.00400
								COW = MCA	0.0280
PDV	Intercept (SA)	0.184	−0.0516	0.378				SA = COW = MCA	0.000999
	COW	−0.984	−1.24	−0.721				SA = COW	0.00400
	MCA	−1.01	−1.29	−0.747				SA = MCA	0.00400
								COW = MCA	0.901
TPStroVol	Intercept (SA) (mean model)	−0.0633	−0.329	0.202				SA = COW = MCA	3.87E-10
	COW (mean model)	1.12	0.792	1.45				SA = COW	7.34E-11
	MCA (mean model)	0.966	0.646	1.29				SA = MCA	1.19E-08
	Intercept (SA) (precision model)	1.21	0.859	1.57				COW = MCA	0.482
	COW (precision model)	0.590	0.125	1.06					
	MCA (precision model)	0.746	0.274	1.22					
TPVFR	Intercept (SA) (mean model)	−0.979	−1.26	−0.696				SA = COW = MCA	0.00131
	COW (mean model)	0.690	0.322	1.06				SA = COW	0.000468
	MCA (mean model)	0.486	0.110	0.862				SA = MCA	0.0274
	Intercept (SA) (precision model)	1.33	0.959	1.70				COW = MCA	0.471
	COW (precision model)	−0.450	−0.910	0.0100					
	MCA (precision model)	−0.434	−0.902	0.0342					
TPVelocity	Intercept (SA) (mean model)	0.465	0.288	0.642				SA = COW = MCA	0.861
	COW (mean model)	0.0137	−0.286	0.313					
	MCA (mean model)	0.0846	−0.224	0.393					
	Intercept (SA) (precision model)	1.60	1.32	1.88					
	COW (precision model)	−0.820	−1.20	−0.436					
	MCA (precision model)	−0.762	−1.16	−0.366					
Area	Intercept (SA)	−2.81	−2.96	−2.66	Intercept: participants	0.135	0.367	SA = COW = MCA	0.000999
	Time	−1.43	−1.67	−1.22	Residuals	2.89	1.70	SA = COW	0.00400
	COW	0.984	0.793	1.15				SA = MCA	0.00400
	MCA	1.08	0.867	1.27				COW = MCA	0.230

**Figure 2:**
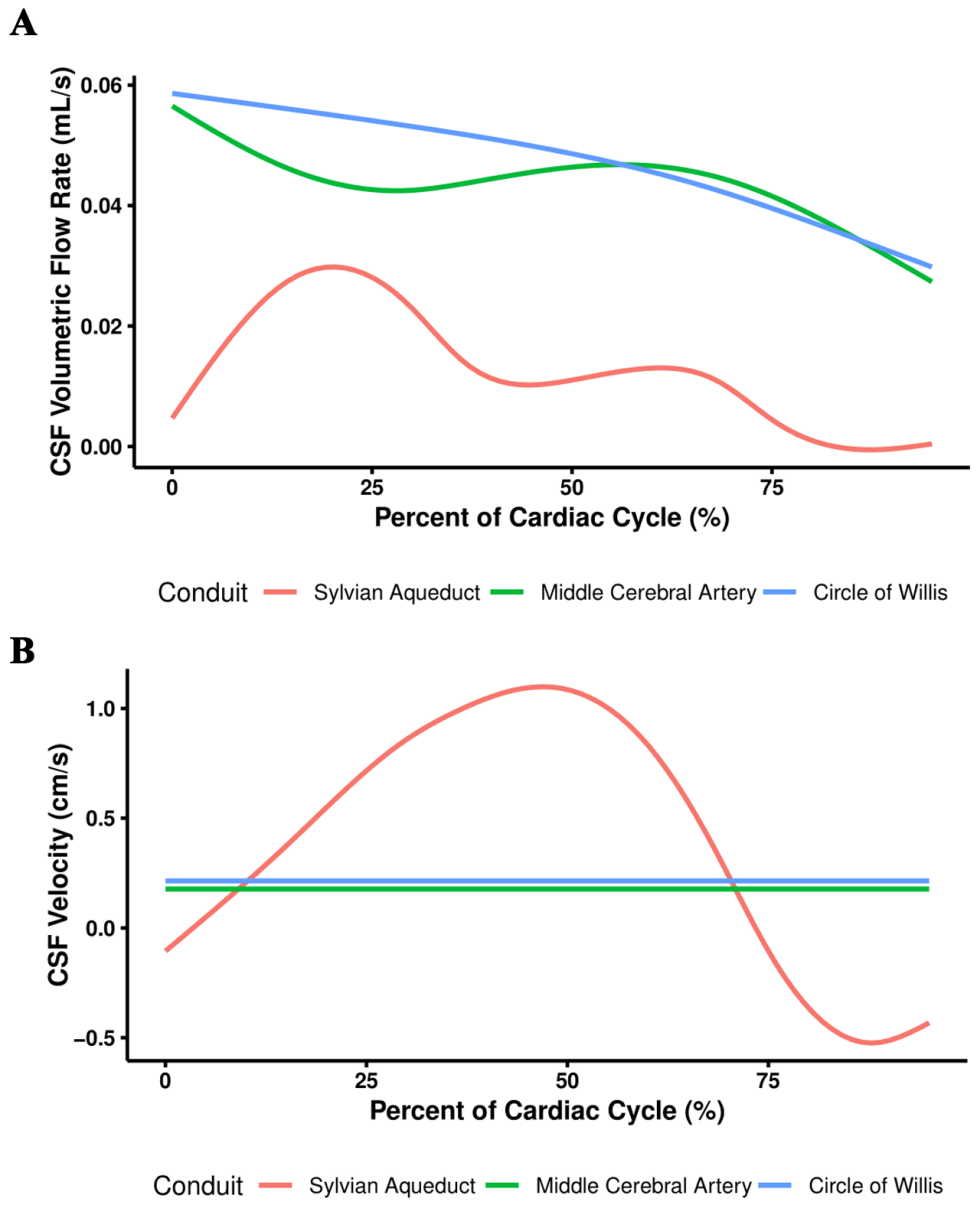
Aggregate CSF flow curves. Aggregation of CSF **(A)** VFR and **(B)** velocity curves for all participants.

As expected, VFR oscillates in the SA which reflects cardiac pulsations. On the other hand, COW VFR decreases throughout the cardiac cycle (Figure 2A). Interestingly, Velocity in the SA is pulsatile while it stays relatively constant in the COW and MCA (Figure 2B).

SA was set as the intercept because CSF flow parameters for this conduit are already established in the literature. The StroVol LMM indicates strong effects with COW StroVol 0.0150 mL (95% CI [0.0103, 0.0190], *p* = 0.00400) and MCA StroVol 0.0142 mL (95% CI [0.00979, 0.0194], *p* = 0.00400) higher than SA (Table 5). The TPStroVol occurs later in the cardiac cycle in COW (*β* = 1.12, 95% CI [0.792, 1.45], *p* = 7.34 × 10–11) and MCA (*β* = 0.966, 95% CI [0.646, 1.29], *p* = 1.19 × 10–8) compared to SA (Table 5).

The LMMs and GLMMs for flow also indicate strong effects of the conduit as the magnitude of the parameters is higher in COW and MCA. Specifically, COW VFR is 0.0350 mL/s (95% CI [0.0261, 0.0460], *p* = 0.00400) and MCA VFR is 0.0310 mL/s (95% CI [0.0203, 0.0411], *p* = 0.00400) higher than SA (Table 5). COW SFR increases by a factor of 1.36 (95% CI [1.20, 1.50], *p* = 0.00400) and MCA SFR by a factor of 1.36 (95% CI [1.21, 1.51], *p* = 0.00400) compared to SA (Table 5). COW DFR also increases by a factor of 1.48 (95% CI [1.16, 1.83], *p* = 0.00799) and MCA SFR by a factor of 1.61 (95% CI [1.33, 1.94], *p* = 0.00799) compared to SA (Table 5). The TPVFR also occurs later in COW (*β* = 0.690, 95% CI [0.322, 1.06], *p* = 0.000468) and MCA (*β* = 0.486, 95% CI [0.110, 0.862], *p* = 0.0274) compared to SA (Table 5).

Velocity in the LMM and GLMs also exhibits strong effects, and the magnitude of the parameters is lower in COW and MCA. COW Velocity is 0.159 cm/s (95% CI [−0.286, −0.0498], *p* = 0.0160) and MCA Velocity is 0.198 cm/s (95% CI [0.0204, 0.0410], *p* = 0.00599) lower than SA (Table 5). COW PSV decreases by a factor of 0.773 (95% CI [−0.954, −0.613], *p* = 0.00400), and MCA PSV decreases by a factor of 0.955 (95% CI [−1.12, −0.773], *p* = 0.00400) compared to SA (Table 5). COW PDV also decreases by a factor of 0.984 (95% CI [−1.24, −0.721], *p* = 0.00400), and MCA PDV decreases by a factor of 1.01 (95% CI [−1.29, −0.747], *p* = 0.00400) compared to SA (Table 5). For TPVelocity, we were unable to find evidence against the hypothesis that SA = COW = MCA (*p* = 0.861) (COW *β* = 0.0137, 95% CI [−0.286, 0.313]) (MCA *β* = 0.0846, 95% CI [−0.224, 0.393]) (Table 5).

For most CSF flow parameters, COW and MCA values are similar (Table 5). It should be noted that the 95% CI and *p*-value (*p* = 0.0280) for PSV contradict each other, but the latter is more directly related to our hypothesis testing (Table 5). Therefore, we found evidence against the hypothesis that COW = MCA for PSV.

There is considerably greater intra-subject variation than between-subject variation for the LMMs and GLMMs which is unsurprising given the pulsatile nature of CSF flow (Table 5). These findings further support our observations in healthy individuals as the effects are comparable.

### 3.1 Sex and age are weak predictors of CSF flow parameters

For all CSF flow parameters, sex, age, and their interaction are on the orders of magnitude less than the intercept (Supplementary Table 1). Moreover, we did not find evidence against the hypothesis that Sex = 0, Age = 0, and their interaction = 0 (Supplementary Table 1). While the 95% CIs and *p*-values of SA DFR (*p* = 0.996), COW DFR (*p* = 0.548), MCA DFR (*p* = 0.999), and MCA TPStroVol (*p* = 0.108) contradict each other, we will defer to the latter as explained previously (Supplementary Table 1). Therefore, we can say with great certainty that the effects of sex, age, and their interaction are weak.

## 4 Discussion

Our results compare favorably with previously reported CSF flow characteristics. We demonstrated pulsatile CSF flow and SA VFR (0.00700 mL/s) in the range of literature values, 0.0049–0.0432 mL/s (Table 2 and Figure 2) (Lee et al., 2004; Wagshul et al., 2006; Yoshida et al., 2009; Oner et al., 2017). However, Flórez et al. (2006) found SA VFR was 0.0635 mL/s in healthy participants. This difference may be attributed to Flórez et al. (2006) use of background correction and their own semi-automated program for creating ROIs. SA Velocity also fell into the reported range of Ståhlberg et al. (1989) study: 0–3 cm/s (Table 2). Overall, these results support the use of our semi-automated program.

SA peak velocities were either similar or lower compared to the literature. SA PSV (1.45 cm/s) and the magnitude of SA PDV (0.867 cm/s) ranged from 2.0 to 11.5 cm/s in the literature (Table 2) (Ståhlberg et al., 1989; Lee et al., 2004; Flórez et al., 2006; Tulupov et al., 2011). Differences may be attributed to the use of tolerance in our semi-automated program and commonly known partial volume effects which cause reduction in maximum flow values, both contributing to underestimated peak velocities (Supplementary Figure 1).

Generally, differences in CSF flow parameters may also be due to differences in MRI manufacturer, artifacts, resolution levels, and VENC (Vandenbulcke et al., 2022). Although CSF velocities observed in our study are low, previous literature has demonstrated that careful selection of low VENC values (e.g., 5 cm/s) in 2D PC-MRI allows for reliable quantification of slow perivascular flow. We acknowledge that novel methods such as IVIM or 4D flow MRI may offer additional insight into global CSF dynamics, but these methods come at the cost of longer scan times and increased complexity. Our goal was to achieve high measurement reliability in targeted anatomical locations which justifies our methodological choice.

While 4D flow MRI provides a comprehensive 3D mapping of CSF dynamics, it is associated with longer acquisition times and increased post-processing demands; in contrast, 2D PC-MRI is a well-validated and reliable method that allows precise and reproducible quantification of CSF velocities in specific anatomical regions of interest. Given the aim of this study is to establish normative reference values in well-defined perivascular regions, 2D PC-MRI was considered the most suitable technique.

What is unique in this study is the thorough examination of CSF flow in the perivascular spaces of COW and MCA. Since our semi-automated program was supported by the SA results, we can now expand its use to measurements of the COW and MCA. For all CSF flow parameters, there is ample evidence they are different in the perivascular spaces of COW and MCA than in the SA (Table 5). Since the magnitude of flow parameters in the COW and MCA is greater while the magnitude of velocity parameters is lower than in the SA, it is plausible the size of these conduits is driving the increased flow rates in the COW and MCA compared to SA (Table 5). The cross-sectional area of the COW and MCA perivascular spaces is larger than SA, so this finding is unsurprising. Between the COW and MCA however, there was minimal difference for most parameters (Table 5). Since fluid flows through the COW and enters the MCA shortly after, this result was expected.

Besides conduits, demographics may also influence CSF flow dynamics. We used our semi-automated program to establish baseline values of CSF flow parameters for each sex and age group. Moreover, we looked at sex, age, and their interaction as predictors. Across the board though, these effects were negligible. Thus, sex and/or age seems to have minimal influence on CSF flow dynamics. Like our study, Sartoretti et al. (2019) used regression models with sex and age as predictors, and they found sex and age could only explain a small part of CSF flow parameters which they quantified to be 6–18%. Furthermore, they found sex and age were not significant predictors for the SA Velocity. Other studies found similar results where sex and age were not significant predictors for the SA VFR, SFR, DFR, Velocity, and Peak Velocity (Flórez et al., 2006; Unal et al., 2009; Oner et al., 2017; Hett et al., 2022).

Some studies, however, have found sex and age dependencies of several CSF flow parameters. Sartoretti et al. (2019) found these predictors were significant for the SA VFR as well as SA peak velocity. The SA VFR and peak velocity increased with age and was higher in males (Sartoretti et al., 2019). Unal et al. (2009) also observed the age dependence of SA peak velocity, but the relationship was inverse. Stoquart-ElSankari et al. (2007) found the age dependence for SA VFR, but similarly, the trend was downward. Rohilla et al. (2023) found weak positive linear correlations with age for the SA SFR and PDV and moderate positive linear correlations for the SA DFR and PSV. This variation in results may be due to the age range of participants and how they were divided into groups. Rohilla et al. (2023) study had participants from 40 to 78 years of age while our study had participants ranging from 23 to 59 years of age. The elderly group in Stoquart-ElSankari et al. (2007) study had a mean age of 71 while the young group had a mean age of 27.5. The difference in CSF flow parameters between these groups may be more obvious because of the higher prevalence of chronic conditions among the elderly. Our study, though, only looked at healthy, relatively young participants.

Our results may have also differed because of our limited sample size. However, Sartoretti et al. (2019) comprehensive study had 128 healthy participants from 17 to 88 years of age which found similar results (Sartoretti et al., 2019). As both of our studies suggest, other factors may influence CSF flow dynamics to a greater extent, including cardiac pulsations, breathing, anatomy of brain, and size of blood vessels (Lee et al., 2004; Sartoretti et al., 2019).

Limitations of our study include eddy currents and partial volume effects. Thus, MRI protocols should be optimized, and the effect of different VENCs on CSF flow parameter measurements should be evaluated. Another major limitation is the inaccuracy of the ROI delineation process. Our semi-automated program used for that process could potentially introduce variability in measurements (Supplementary Figure 1). Furthermore, it is only capable of capturing one continuous ROI. Thus, if conduits appear in multiple areas of the MRI phase image as in the case of the COW and MCA, measurements would be underestimated. To improve the ROI delineation process, our semi-automated program should be formally evaluated, and its inter-observer reliability should be measured (Supplementary Figure 1). We believe these efforts would be beneficial as automation has the benefits of increasing accuracy, reproducibility, and the ease of studying large samples (Hett et al., 2022). Lastly, it is clear there is no consensus on the effects of sex, age, and their interaction, even in literature on the SA. Further studies, then, need to be conducted and particularly focus on comparing the elderly population to younger populations.

In this study, we have established for the first time the baseline values for the perivascular spaces of COW and MCA CSF flow parameters and compared them to those in the SA. We also highlighted the limited influence of sex and/or age. Future studies can use this research as a starting point to investigate the CSF flow in the perivascular spaces of COW and MCA, thereby increasing the accuracy of parameter measurements. It might also be helpful to look at sex, age, and other factors (e.g., breathing) simultaneously to get a better sense of what drives CSF flow dynamics. Finally, these studies should be repeated in patients with central nervous system or cerebrovascular system pathologies which could potentially lead to the applications of CSF flow to clinical diagnosis, monitoring, and treatment.

## Data Availability

The original contributions presented in the study are included in the article/Supplementary material, further inquiries can be directed to the corresponding authors.
